# Evaluating the Potency of Selected Antibiotic Medications Dispensed in Community Pharmacies in Gwale, Kano, Nigeria

**DOI:** 10.3390/antibiotics12111582

**Published:** 2023-10-31

**Authors:** Muhammad Dauda Mukhtar, Fatihu Ahmad Rufa’i, Abdurrahaman Umar Yola, Nafisa Ibrahim Babba, Daniel Baecker

**Affiliations:** 1 Department of Pharmaceutical Microbiology and Biotechnology, Faculty of Pharmaceutical Sciences, Bayero University Kano, Gwarzo Road, Kano PMB 3011, Nigeria; abdrrahmanyola@gmail.com (A.U.Y.); nafisababba89@gmail.com (N.I.B.); 2 Kano Liaison Office, Nigerian Institute for Trypanosomiasis (and Onchocerciasis) Research, Surame Road, Kaduna PMB 2077, Nigeria; fatihurufai@gmail.com; 3 Department of Pharmaceutical and Medicinal Chemistry, Institute of Pharmacy, Freie Universität Berlin, Königin-Luise-Straße 2+4, 14195 Berlin, Germany

**Keywords:** antibiotics, *Clostridium tetani*, counterfeit medications, disc-diffusion assay, drug quality, *Escherichia coli*, Nigeria, resistance, *Staphylococcus aureus*

## Abstract

The worsening of antibiotic resistance is a multifactorial process. One aspect of this is the counterfeiting of antibiotic medications. This is supposed to be particularly high in developing countries, including Nigeria. Therefore, the potency of some antibiotic drugs dispensed in community pharmacies in Gwale, Kano, Nigeria, was investigated in this case study. Three products, each from different manufacturers, with the active ingredients of ceftriaxone, gentamicin, ciprofloxacin, and metronidazole, respectively, were included in this study. By means of a disc-diffusion assay, the effect against the typed strains *Staphylococcus aureus* (ATCC 25923) and *Escherichia coli* (ATCC 25922) as well as *Clostridium tetani* isolated from soil was tested. Clinical isolates of *S. aureus* and *E. coli* were also used. While antibiotics, with the exception of ciprofloxacin-containing preparations against *C. tetani*, showed acceptable efficacy against the typed strains by comparison with the clinical science laboratory references, a predominant failure was observed with the clinical isolates. Thus, the investigated drug preparations can be considered of acceptable quality for the treatment of susceptible bacterial infections. This excludes counterfeits in the sampled preparations. However, the insufficient efficacy against clinical isolates further documents the severity of nosocomial bacteria.

## 1. Introduction

Substandard and counterfeit drugs are a serious public health problem, especially in developing countries [1]. In these countries, including Nigeria, there is growing concern about the efficacy of antimicrobials. It was reported in Nigeria that certain antibiotics are of poor quality [2].

Antibiotics are the most commonly counterfeited drugs, accounting for 28% of global faked pharmaceuticals. No area of the world seems to be spared from antibiotic counterfeiting [3]. The sale of falsified medicines accounts for 6% of the global market, with some regions at their worst [2].

It was indicated that 70% of medicines in Nigeria in the year 2002 were substandard or counterfeit [4], while the National Agency for Food, Drug Administration, and Control (NAFDAC) estimated that only 41% were counterfeit in 2001, recording a further decline [5]. However, the counterfeiting of antibiotics, in any case, contributes to the worsening of the resistance situation.

Antimicrobial resistance is a growing public health problem worldwide due to a variety of factors, including the poor quality of antibiotic medications [6]. In view of this, the present case study was undertaken to evaluate the potency of some parenteral antibacterial preparations dispensed in community pharmacies in Gwale, Kano, Nigeria.

## 2. Results

The antibacterial activity of selected medications dispensed in community pharmacies in Gwale was checked using the drug diffusion test. The values obtained were compared with values of the Clinical and Laboratory Standard Institute (CLSI) for the ATCC strains *S. aureus* and *E. coli* [7]. The results against non-clinical isolates of *S. aureus* and *E. coli*, as well as against *C. tetani* isolated from soil, are given in Table 1.

From the three preparations containing the drug ciprofloxacin, discs were loaded with 10 µg of the antibiotic for the disc-diffusion test. In the assay, all three were active against *S. aureus*, displaying an inhibition zone diameter of 25–27 mm. By comparison with the CLSI breakpoint values for ciprofloxacin (22–30 mm), the antibacterial potency requirements were met. The same was the case for the effect on *E. coli*. The diameter of the zone of inhibition was 33–36 mm (CLSI: 29–38 mm). In contrast to these two Gram-negative bacteria, the activity against the Gram-positive *C. tetani* failed. A diameter of 17–20 mm was measured, thus falling below the reference value (26 mm). It is noteworthy that the ciprofloxacin-containing products of all three manufacturers showed a very similar potency among themselves. This indicates the uniform content of the active ingredient in the three preparations from different producers or suggests equal bioavailability of the drug despite the different composition of preparations.

The mean diameters of the inhibition zones caused due to the treatment of bacteria with ceftriaxone (30 µg) confirmed the efficacy of the drug. In particular, it was 20–36 mm against *S. aureus* (CLSI: 22–28 mm), 36–38 mm against *E. coli* (29–35 mm), and 21–23 mm against *C. tetani* (reference: 20 mm). The three preparations containing ceftriaxone showed a very balanced profile among themselves against *E. coli* and *C. tetani*. Preparation D (36 mm) was striking against *S. aureus*. It had a considerably larger zone of inhibition than the other two preparations, E (23 mm) and F (20 mm). A better bioavailability of the antibiotic drug in the case of preparation D compared to the differently composed preparations E and F could be responsible for this but was not further investigated in this study.

The three gentamicin samples showed activity against *S. aureus*. The diameters of their inhibition zones were coherently 21–23 mm, which covers the value of CLSI (19–27 mm). Against *E. coli* (CLSI: 19–26 mm), products G (23 mm) and H (26 mm) passed. Surprisingly, product I (0 mm) showed no activity at all. Again, pharmacokinetic aspects such as the release of the antibiotic drug from the dosage form could be considered a reason for this due to the different composition of preparations. However, all three preparations passed when treating *C. tetani* (25–26 mm; reference: 16 mm).

The metronidazole-containing drugs were not studied against the facultatively anaerobic bacteria *S. aureus* and *E. coli* but against the obligately anaerobic *C. tetani*. They were active, exhibiting diameters of 19–26 mm (reference: 18 mm).

The activity of antibiotics against the clinical isolates of *S. aureus* and *E. coli* was also determined using the disc-diffusion assay. The results are shown in Table 2.

The diameter of the zone of inhibition was 18–26 mm when the clinical isolate of *S. aureus* was treated with ciprofloxacin (10 µg). Compared to the value of the CLSI (≥21 mm), two preparations (product A: 20 mm, product C: 18 mm) did not reach the threshold and were, therefore, considered to have failed. Product B (≥26 mm) passed. When treating the clinical isolate *E. coli*, the same pattern was observed between the three different ciprofloxacin-containing antibiotics. Product B (26 mm; CLSI: 26 mm) was effective, whereas product A (25 mm) and product C (25 mm) were, very narrowly, not. The difference to preparation B could again be the different composition of medications from different manufacturers. However, this was not further investigated in the current study.

Also, the medications with ceftriaxone were, for the most part, not as active against clinical isolates. Against *S. aureus*, product D (24 mm; CLSI ≥ 20 mm) showed sufficient potency. Product E (15 mm) and F (14 mm) were clearly less active. Product F (0 mm) strikingly exhibited no activity at all against *E. coli*. This could be due to pharmacokinetic issues with preparation F. Product D (19 mm; CLSI: ≥20 mm) failed by a small margin, but product E (34 mm) was potent.

The diameters of the zone of inhibition when gentamicin (10 µg) was used against the clinically relevant *S. aureus* strain were 12–16 mm (CLSI: ≥15 mm). Two medical preparations (product G: 12 mm, product I: 12 mm) did not meet the reference value (≥15 mm), and only product H (16 mm) was successful. Against *E. coli* from the clinics, antibiotics G (21 mm) and H (20 mm) exceeded the standard control (≥16 mm). However, preparation I (0 mm) failed totally. Such a failure as that of product I was already observed with the ATCC strain. This suggests a general issue with preparation I itself compared to the preparations of other manufacturers.

Overall, data suggest that when the drugs were tested on clinical bacterial isolates, they showed much higher failure rates than the typed bacteria. The low susceptibility of the isolates obtained from the hospital once again documents the problem of the resistance of nosocomial microorganisms.

## 3. Discussion

From the perspective of drug counterfeiting, the present study aimed to evaluate the efficacy of some parenteral antibacterial agents dispensed in public pharmacies in Gwale, the local government area of Kano, Nigeria. The WHO generally recommends the post-marketing surveillance of essential medicines even after marketing or at their dispensing point [8]. These also include antibiotics. The subject of this investigation was intravenous formulations with the active ingredients ciprofloxacin, ceftriaxone, gentamicin, and metronidazole, respectively, from three different manufacturers each. The expenses for all formulations used in this study were almost the same. Therefore, no influence as to costs on the efficacy of the formulations could be derived. The bacteria used were ATCC strains and clinical isolates of *S. aureus* and *E. coli*, as well as a *C. tetani* strain isolated from soil. The microbiological disc-diffusion test was used to evaluate antibacterial efficacy. This assay is the most widely used test for routine antibiotic or bacterial susceptibility testing. As a result, the diameter of a zone can be obtained in which no growth of the microorganism occurs due to the diffusion of the antibiotic into the agar. The obtained values were compared with those of the control standard of CLSI.

Based on the results of the typed strains, previous claims about the general prevalence of counterfeit drugs were refuted. In the vast majority of cases, potency was demonstrated. The antibacterial activity of the three brands of ceftriaxone (30 µg) against *S. aureus* (ATCC 25923), and *E. coli* (ATCC 25922), as well as the soil isolates of *C. tetani* had no statistical difference and, compared with the control standard of the CLSI, the drugs passed the tests.

Ciprofloxacin (10 µg) passed the test at 100% with a range of 25–27 mm against *S. aureus* (*p* = 0.667) and 33–36 mm against *E. coli* (*p* = 0.27). However, 100% failed with 17–20 mm against *C. tetani* when isolated from soil. This does not agree with the data of Bukar et al. [9], who reported a value of 26 mm for *C. tetani*.

Gentamicin (10 µg) also passed the test at a level of 21–23 mm against *S. aureus* (*p* = 0.225) and against soil *C. tetani*. Against *E. coli* (*p* = 0.531), one of the three preparations was completely inactive.

However, the predominant inactivity of ciprofloxacin, ceftriaxone, and gentamicin against bacterial clinical isolates of *S. aureus* and *E. coli* documented poor susceptibility and indicated resistance. This is in good agreement with a similar study in Ibadan, Nigeria, performed by Alabi and Ijose [10]. At the international level, the present results are also comparable with the reports of Gashe et al. [11] from Ethiopia and Wu et al. [12] from China. The full activity of metronidazole-containing preparations against *C. tetani* in wild soil is also consistent with reports from a study by Bukar et al. [9].

Although the natural phenomenon of resistance formation is accelerated and amplified by a variety of factors [13], one of the most important causes is the improper use of antimicrobial agents. Compared to the standard control organisms (ATCC) investigated under identical conditions, the results of the susceptibility test, which used clinical isolates instead, showed how all bacterial isolates used in this study seemed to be multidrug-resistant strains.

Nevertheless, minor variations in the efficacy of some brands of parenteral antibiotics used in this study against the same organism were also observed. This is not addressed further in this study, but it could be a result of differences in the formulation methods and the excipients used. These may consequently influence the penetration of antibiotics into the bacterial cell. Furthermore, this could be due to storage conditions [14]. Indeed, these could affect the efficacy and overall quality of the medications. Even if it is assumed that the medicines were properly stored when purchased from pharmacies, this information would have to be checked for unequivocal verification. This was not conducted in the current investigation and could be seen as a limitation of this study.

Considering the positive results against the typed bacterial strains, it can be assumed in this study that parenteral antibacterials dispensed in community pharmacies in Gwale did not show signs of counterfeiting. Therefore, the studied drug preparations can be considered of acceptable quality for the treatment of susceptible bacterial infections. However, these potent antibiotics did not show activity against the clinical bacterial isolates due to the occurrence of resistance.

This further demonstrates that the resistance situation is a serious problem. To address this issue in the future, the emergence of resistance mechanisms needs to be further elucidated [13], as well as their persistence and spread. Novel potential modes of antibacterial action have to be discovered [15]. In addition, in the so-called post-antibiotic era, the development of new drug candidates, for example, via extraction from plants [16], is desirable in the near future.

Moreover, when prescribing antibiotics, clinicians should actually base their choice on scientific evidence (e.g., as a result of an antibiogram). Overall, promoting the responsible use of antibiotics and raising public awareness of the threat of antibiotic resistance is recommended [17]. Ultimately, preventing the spread of infection reduces the need for antibiotics, which, in turn, decreases resistance. Agencies involved in regulating the sale of medicines should also ensure in the future that pharmacies display and store their medicines under proper storage conditions, especially antibiotics, which require strict storage conditions. Moreover, strategies to monitor the efficacy of antibiotic medicines and to eradicate counterfeit or substandard medicines should be intensified. However, the present study has made a corresponding contribution to this. It can serve as an example for other countries in the world than just Nigeria. After all, the WHO acts worldwide with its recommendations, and the antibiotic resistance situation is a global problem.

## 4. Materials and Methods

### 4.1. Collection and Preparation of Bacterial Samples

The bacterial isolates *Staphylococcus aureus* (ATCC 25923) and *Escherichia coli* (ATCC 25922) were obtained from the Department of Microbiology, Bayero University Kano, Nigeria. Wild local *Clostridium tetani* was isolated from soil in Bayero University Kano, and identified, and confirmed in the microbiology laboratory of Bayero University Kano, Nigeria. The inclusion of *C. tetani* in the bacteria studied is justified by an elevated prevalence of tetanus (about 75%) in the study area. Clinical isolates of *E. coli* and *S. aureus* were collected from patients attending the Aminu Kano Teaching Hospital Kano. Colonies of bacteria were sub-cultured in fresh nutrient agar, and their morphological appearance monitored via microscopy was adduced for the purpose of identification. In addition, bacteria were characterized by Gram staining and biochemical tests as described in the literature [18].

### 4.2. Antibiotic Drugs

Four antibiotic compounds with different mechanisms of action were selected for this study. These drugs were ceftriaxone, gentamicin, ciprofloxacin, and metronidazole. Ceftriaxone is a third-generation cephalosporin antibiotic. As a member of the β-lactams, it inhibits bacterial cell wall synthesis. Gentamicin is an aminoglycoside antibiotic that interferes with bacterial protein biosynthesis. Ciprofloxacin belongs to the class of fluoroquinolones and inhibits bacterial topoisomerase II (gyrase). Metronidazole is a nitroimidazole acting against anaerobic microorganisms. It interferes with nucleic acid synthesis through reactive species formed as a result of reduction processes.

The subjects of this study included three different medical products (different brands) for the intravenous use of each of the three antibiotics. The composition of the medications is described as follows: preparation (A) Ciprotab (34 mg of ciprofloxacin, 5 mg of povidone, 7 mg of hydroxypropyl methylcellulose (hypromellose), 12 mg of magnesium stearate, and 10 mg of cellulose), preparation (B) Cipro (38 mg of ciprofloxacin, 3 mg of lecithin, 4 mg of polysorbate, 11 mg of polyethylene, and 20 mg of maize starch), preparation (C) Ciloxan (67 mg of ciprofloxacin, 8 mg of titanium dioxide (E171), 6 mg of starch glycolate, 40 mg of macrogol, and 13 mg strawberry flavor), preparation (D) Rocephin (12 mg of ceftriaxone, 7 mg of corn stick liquor, and 12 mg of sucrose), preparation (E) Ceftriaxone (1 mg of ceftriaxone sodium salt and 21 mg of starch), preparation (F) Ceftriaxone-N (12 mg of ceftriaxone and 12 mg of starch), preparation (G) Gentafair (12 mg of gentamicin sulphate, 30 mg of tyloxapol, and 21 mg of propylene glycol), preparation (H) Gentasol (12 mg of gentamicin, 10 mg of glycerol monostearate, 12 mg of isopropyl myristate, and 3 mg of sorbitol solution), preparation (I) Gentatak (10 mg of gentamicin, 30 mg of sodium hydrochloride, and 21 mg of polyethylene glycol), preparation (I) Flagyl (19 mg of metronidazole, 9 mg of corn starch, and 10 mg of black iron oxide), preparation (K) Flagyl ER (20 mg of metronidazole, 12 mg of titanium dioxide, 8 mg of sodium salt solution), preparation (L) Flagyl RTU (12 mg of metronidazole and 7 mg of magnesium stearate). All medications were obtained from reputable pharmacies in Gwale (a local government area in Kano State, Nigeria) between December 2022 and March 2023. When purchasing medications in pharmacy stores, the correct storage according to the regulations was agreed upon. The pharmacopeial details, e.g., brand, dosage, manufacturer, etc., were identified and recorded accordingly. All tested formulations had a certificate from the National Drug Control Agency.

### 4.3. Preparation of Antibiotic Discs for Disc-Diffusion Test

Whatman filter paper discs with a diameter of 6.0 mm were punched out with a hole punch and heat-sterilized in Bijou screw cap bottles for each drug brand at 140 °C in an oven for 1 h and then cooled to room temperature. The solution of antibiotics was prepared using dimethyl sulfoxide (DMSO) as a diluent. The discs were impregnated with the antibiotic solutions so that 1.0 mL contained 100 times the amount of active ingredient required for each disc of the different drugs. The final amount of the drug accounted for 10 µg (ciprofloxacin, gentamicin, and metronidazole) or 30 µg (ceftriaxone). The discs were stored at 25 °C under wet conditions before use [2].

### 4.4. Standardization of the Bacterial Inoculum

Subcultures of *C. tetani*, the ATCC strains of *E. coli* and *S. aureus*, as well as their clinical isolates, were made by carefully picking one colony with a sterile inoculation loop, followed by inoculation on the surface of a nutrient agar slant for 24 h. Using a sterile swab stick, the overnight nutrient broth culture was diluted with saline so that the turbidity was adjusted to the 0.5 McFarland standard. This was established to result in bacterial suspension with a mean of 3.3 × 10^6^ colony-forming units (CFU)/mL for microbial population density. The inoculum was determined by comparison with the turbidity of a solution of barium sulfate (1%, *v*/*v*), as described and performed before [19].

### 4.5. Procedure of the Disc-Diffusion Test

A sterile standard swab was dipped into a suspension of the prepared inoculum solution. Next, the swab was swabbed uniformly and aseptically over the surface of the Mueller Hinton agar plate, rotating the plate to ensure an even distribution. The prepared antimicrobial discs were then aseptically pressed down with forceps. After 10 min of pre-diffusion, the plates were incubated in a microbiological incubator at 37 °C for 18 h. The diameters of the inhibition zones (reports in mm) were measured in replicates using a Vernier caliper. The data are given as the mean ± standard deviation of *n* = 6 independent experiments [19].

### 4.6. Statistical Data Analysis

The activities of each drug on ATCC-susceptible isolates were expressed as the mean ± standard deviation of *n* = 6 independent experiments. Statistical significance was considered at *p* ≤ 0.05.

## Figures and Tables

**Table 1:** Antibacterial activities of drugs from intravenous medications dispensed in community pharmacies in Gwale against typed bacteria. Data represent the mean ± standard deviation of *n* = 6 independent experiments.

Antibiotic	Brand Name	Disc Potency (µg)	Diameter of the Zone of Inhibition (mm)		
			*S. aureus * (ATCC 25923)	*E. coli * (ATCC 25922)	*C. tetani* (Isolated from Soil)
Ciprofloxacin	Ciprotab (A)	10	25.00 ± 1.32	36.01 ± 1.10	20.00 ± 1.75
	Cipro (B)		27.00 ± 0.12	36.08 ± 4.80	17.50 ± 0.50
	Ciloxan (C)		25.31 ± 0.27	33.02 ± 2.60	18.00 ± 0.28
	Standard		22–30	29–38	26
Ceftriaxone	Rocephin (D)	30	36.01 ± 2.82	38.30 ± 4.10	22.00 ± 0.59
	Ceftriaxone (E)		23.08 ± 1.18	38.00 ± 0.51	21.02 ± 0.05
	Ceftriaxone-N (F)		20.87 ± 0.22	36.00 ± 1.00	23.00 ± 0.05
	Standard		22–28	29–35	20
Gentamicin	Gentafair (G)	10	21.03 ± 0.11	23.00 ± 0.49	26.00 ± 1.16
	Gentasol (H)		22.00 ± 0.35	26.04 ± 0.57	26.00 ± 1.02
	Gentatak (I)		23.90 ± 0.27	0.00 ± 0.00	25.05 ± 1.25
	Standard		19–27	19–26	16
Metronidazole	Flagyl (J)	10	-	-	19.08 ± 1.09
	Flagyl ER (K)		-	-	22.04 ± 2.19
	Flagyl RTU (L)		-	-	26.00 ± 0.68
	Standard				18

**Table 2:** Antibacterial activities of drugs from intravenous medications dispensed in community pharmacies in Gwale against bacteria isolated in the hospital. Data represent the mean ± standard deviation of *n* = 6 independent experiments.

Antibiotic	Brand Name	Disc Potency (µg)	Diameter of the Zone of Inhibition (mm)	
			*S. aureus* (Clinical Isolate)	*E. coli* (Clinical Isolate)
Ciprofloxacin	Ciprotab (A)	10	20.00 ± 0.58	25.00 ± 1.90
	Cipro (B)		26.00 ± 0.36	26.00 ± 0.51
	Ciloxan (C)		18.10 ± 0.08	25.00 ± 1.11
	Standard		≥21	≥26
Ceftriaxone	Rocephin (D)	30	24.00 ± 5.37	19.00 ± 0.56
	Ceftriaxone (E)		15.60 ± 0.50	34.00 ± 1.48
	Ceftriaxone-N (F)		14.00 ± 0.09	0.00 ± 0.00
	Standard		≥20	≥20
Gentamicin	Gentafair (G)	10	12.20 ± 0.10	21.00 ± 0.49
	Gentasol (H)		16.00 ± 0.30	20.30 ± 0.45
	Gentatak (I)		12.00 ± 0.49	0.00 ± 0.00
	Standard		≥15	≥15

## Data Availability

The data presented in this study are available in the manuscript.
